# The Active Waiting Standard: turning the referral gap into a managed phase of care

**DOI:** 10.3389/fmed.2026.1800471

**Published:** 2026-04-13

**Authors:** Waseem Jerjes, Azeem Majeed

**Affiliations:** Department of Primary Care and Public Health, Faculty of Medicine, Imperial College London, London, United Kingdom

**Keywords:** Active Waiting Standard, care transitions, clinical responsibility, integrated care, patient safety, primary care continuity, referral pathways, waiting lists

## Introduction

Primary care clinicians and patients recognize a familiar sequence: a referral is sent, the receiving service acknowledges it, and the patient enters a waiting list. During the interval between “referral sent” and “first specialist contact,” symptoms may progress, medicines are frequently escalated, function and sleep deteriorate, and anxiety rises. Administratively, this interval is often treated as “inactive time”: the referral has been logged and the wait has started. Clinically, however, this is not inactive time. It is an unstructured phase of care that may carry preventable risk.

This is not a marginal phenomenon. In July 2025, England reported 7.4 million RTT (referrals to treatment) pathways open across around 6.2 million people; only 61.3% were within 18 weeks and many waits extended well beyond months (1). The 18-week standard has not been met for years, and while the waiting list has reduced modestly from its late-2023 peak, it remains historically high (2). For many patients, “waiting” is not a short administrative interlude; it is a common lived experience of care.

The impact is felt most immediately in general practice. In the 12 months to June 2025, GPs delivered a record 383.3 million appointments (3). This reflects a sustained shift of uncertainty management, risk containment, and patient support into primary care while specialist access is delayed. Yet governance and measurement still leave the referral-to-first-contact interval largely without clear ownership, explicit standards, or shared escalation logic. In effect, the system measures the wait but does not systematically manage the risks of waiting.

There are signals that the system is beginning to “see” the problem more clearly. National publication of waiting list breakdowns by age, sex, ethnicity, and deprivation improves visibility of who waits longest, and potentially who is more exposed to harm while waiting (4). However, visibility alone does not make waiting safer. If the interval remains unowned—without accessible contact routes, baseline assessment, scheduled check-ins, and explicit triggers for action—avoidable deterioration and unplanned care will continue to accumulate. Although the evidence cited here is drawn from England, prolonged elective waits and fragmented accountability during the waiting period are widely recognized across health systems; the principles proposed are therefore transferable beyond a single national context.

This Opinion argues for treating the referral-to-first-contact interval as a named, managed phase of care and proposes a practical, auditable minimum bundle—the Active Waiting Standard (AWS)—to convert “waiting time” into “safer time.” AWS is intentionally designed as a minimum safety specification rather than a new service: each component can be recorded, monitored, and reviewed, making the quality of “waiting” visible rather than assumed. The aim is constructive: to offer an implementable framework capable of reducing harm and inequity while patients wait.

### The referral-to-first-contact interval is a phase of care, not a queue

Elective systems commonly conceptualize waiting lists as queues, where the primary performance variable is time-to-appointment. Clinically, the variable that matters is trajectory: whether a patient remains stable, deteriorates, develops complications, or experiences avoidable risk while waiting. When the interval is treated like a queue, care often becomes reactive: contact occurs only when symptoms escalate, medicines drift, or a crisis forces urgent access. A phase-of-care approach instead treats the interval as an expected stage requiring minimum safety standards.

A queue model also creates an accountability problem. Patients often assume that once a referral is accepted, responsibility has transferred. In practice, the receiving service has not assessed the patient and has not assumed clinical responsibility in a meaningful sense. The result is a “gray zone”: no named lead, uncertain contact routes, unclear thresholds for escalation, and no explicit point at which clinical responsibility is confirmed as having transferred. Patients may be left unsure whether to re-contact the GP, chase the hospital, or simply endure worsening symptoms. Clinicians may be uncertain when escalation will succeed, what baseline data matters, and how to document change in a decision-relevant way.

Professional standards already point toward closing this gap. Good Medical Practice emphasizes effective information sharing, clarity about who is responsible for which aspects of care, and ensuring that responsibility has actually been accepted at transitions (5). The referral-to-first-contact interval functions like a transition without a “handshake”: the referral has been acknowledged, but clinical takeover has not occurred. This is not merely an inconvenience; it is a patient-safety vulnerability created by ambiguous ownership.

A practical response is to make ownership explicit and time-limited. In the AWS model, the Named Guardian would usually be the referring GP, because this clinician is most likely to know the patient's baseline, referral rationale, and evolving treatment context at the point the waiting period begins. Where pathways are deliberately team-based, the role could instead be assigned to another appropriately skilled clinician within the practice or neighborhood team, provided that responsibility is explicit, contactable, and maintained until the receiving service confirms clinical takeover. This does not imply that primary care should substitute for specialist care. Rather, it recognizes that the system should stop treating the interval as unowned time. Ownership becomes visible, contactable, and auditable.

This approach also aligns with system priorities. National operational planning guidance for 2025/26 emphasizes reducing waits, improving triage, better use of advice-and-guidance, waiting list validation, and reducing avoidable demand (6). A Named Guardian standard translates these priorities into a concrete local bundle: clear contact routes, risk-banded follow-up, and pre-defined escalation pathways designed to minimize deterioration and improve the value of first specialist contact.

### The Active Waiting Standard: a practical A–E bundle for safer waiting

The Active Waiting Standard (AWS) is a minimum, auditable bundle designed for the period between referral and first specialist contact. It does not depend on unpublished data, specialist substitution, or complex infrastructure. It focuses on five essentials—an A–E structure that is easy to teach, implement, and measure:

#### A—Access (and visible ownership)

Patients are told who their Named Guardian is during the wait—usually the referring GP unless another appropriately skilled clinician has been explicitly designated—and how to make contact. A single clearly signposted route is provided (e.g., telephone line plus message route), with a defined response standard and recorded consent for carer involvement where appropriate. Access must be inclusive: non-digital routes should exist alongside digital routes from the outset, because digital exclusion clusters with higher-risk contexts such as older age, deprivation, disability, and language barriers. A national framework emphasizes designing services for inclusion rather than treating non-digital access as an exception (7). Population data on adults' media use further reinforces persistent variation in digital access and capability (8). If AWS is to reduce inequity, inclusive access must be a core standard rather than a local add-on.

#### B—Baseline (making change visible)

On the day of referral—or soon after—the Named Guardian captures a brief, decision-relevant baseline and a short patient-reported statement. The goal is not data collection for its own sake, but making later change visible: symptom intensity, key function prompts, and relevant risk flags. A minimal baseline improves safety-netting and makes escalation more credible because deterioration can be evidenced rather than described vaguely. Baseline elements should be condition-specific but light-touch: what can realistically be captured without turning waiting into a parallel clinical programme.

#### C—Check-ins (risk-banded and light-touch)

Using the baseline and clinical context, the patient is offered a risk-banded schedule of check-ins. For low-risk cases, check-ins can be infrequent and largely patient-reported; for higher-risk contexts, more frequent structured check-ins are warranted. The key design principle is signal-triggered review: short patient-reported prompts (digital or non-digital equivalents) that trigger clinician review when a threshold is crossed (worsening symptoms, functional decline, medication escalation, new safeguarding concerns). This reframes elective pathways as dynamic rather than static while patients wait.

#### D—Defined escalation (pre-agreed triggers and routes)

AWS requires explicit escalation thresholds and pre-planned routes, such as advice-and-guidance, accelerated messages to the receiving service, community diagnostics, urgent response pathways, or urgent care when appropriate. This is where AWS interfaces with elective reform. National elective care reform emphasizes advice-and-guidance, triage, and pathway design (9). AWS operationalises that approach during the waiting period: escalation becomes expected, structured, and justifiable when trajectory changes.

#### E—Essentials for self-care (the “Wait Plan”)

AWS includes a one-page “Wait Plan” that explains: what is being waited for and why today is safe; what is being monitored; how to make contact; what triggers urgent action; and practical condition-relevant self-care steps. The Wait Plan must be available in translated and paper formats by default to avoid embedding inequity into access. It also creates psychological containment: patients can understand what is happening, what to do, and what will happen if they worsen.

Although AWS is diagnosis-agnostic, implementation is helped by illustrating the bundle in high-volume streams (Table 1). For osteoarthritis, evidence-based recommendations emphasize conservative management and avoiding low-value imaging; a baseline plus clear self-care plan supports safe holding care and avoids investigation drift (10). For chronic heart failure awaiting clinic review, baseline tracking and explicit escalation thresholds are central to avoiding decompensation; established recommendations support structured monitoring and safety-netting while patients wait (11). These examples show how AWS does not replace specialist care; it reduces risk and improves readiness for the specialist encounter.

**Table 1:** Active Waiting Standard (A–E): minimum bundle by common referral category and expected effects during the waiting period.

Referral category	A: Access and ownership	B: Baseline (what we track)	C: Check-ins (when/how)	D: Defined escalation (act now if…)	E: Essentials for self-care (starter steps)	Expected effect while waiting
MSK (e.g., knee OA)	Named Guardian recorded; single contact route; response within 2 working days; non-digital route available; carer consent recorded	Pain 0–10; simple function prompt	Weeks 2, 6, 10; short PROM-style prompts with call if signal	New neurological deficit; red-flag symptoms; pain ≥8/10 for ≥72 h despite step-up	Pacing/strengthening plan; topical options where appropriate; sleep routine; physiotherapy pathway information	Less unscheduled care; clearer escalation evidence; improved first-visit value
Moderate mental health	Named clinician; crisis route signposted; carer involvement recorded	Brief symptom score; sleep quality; risk screen	Weeks 1, 3, 6; phone/message/paper-equivalent	Any suicidal ideation; marked score rise; self-neglect or safeguarding risk	Self-care pack; sleep resources; social support signposting	Earlier risk detection; fewer crises; improved function while waiting
Suspected cancer (downgraded)	Direct contact route held by Named Guardian; clear plan for re-escalation	Symptom diary; weight; red-flag checklist	Weeks 2, 6; short phone review	Any new red flag; unintentional weight loss; clear adverse trajectory	Analgesia plan; nutrition advice; written escalation card	Maintained vigilance; faster re-escalation when trajectory changes
Chronic pain	Named GP/pharmacist access; response within 2 working days	Pain interference; current analgesic load	Weeks 2, 6, 10; brief prompts; pharmacist review if signal	Escalating sedating meds/opioids; functional decline; adverse effects	Non-drug pacing; movement “snacks”; sleep routine	Reduced medication drift; fewer urgent contacts; improved coping
Heart failure (awaiting clinic)	Named Guardian; urgent route for red flags	BP/HR; daily weight; symptom class	Weeks 1, 3, 6; phone; earlier if signal	Weight rise >2 kg in 3 days; resting breathlessness; syncope/chest pain	Daily weights; salt/fluid guidance; explicit stop-rules	Fewer decompensations; safer holding care; better specialist readiness

### Measuring harm, not just time: building a “Wait Harm” view

Waiting is usually described in weeks. This tells us how long people spend on a waiting list, but little about what happens to them during that time. Length alone is a poor proxy for risk. Clinically, what matters is whether people deteriorate while waiting, whether they require unplanned care, and whether function, mental health, or social stability erodes in ways that are difficult to reverse.

A meaningful “Wait Harm” view can be built largely from information already present in primary care records combined with brief patient reporting. Indicators include symptom trajectories, functional decline, prescribing drift (especially escalation or initiation of higher-risk medicines), and unplanned care episodes. Social markers—loss of work, increased carer burden, reduced ability to self-care—capture harms that seldom appear in referral letters but meaningfully affect outcomes and recovery.

Equity must be visible within the same time horizon. Commentary on new waiting list breakdowns highlights that inequality is not simply about who waits longest, but who experiences the greatest harm while waiting. The King's Fund has emphasized the importance of the new data for understanding and tackling inequalities within waiting lists (12). Nuffield Trust analysis has also described disparities in waiting times between patient groups as deeply concerning (13). If AWS is adopted, stratified “Wait Harm” metrics (by age, sex, ethnicity, deprivation) would allow systems to identify where harms cluster and to apply proportionate mitigations: non-digital routes, translated materials, proactive check-ins, and carer-inclusive communication.

Continuity is a core harm-reduction mechanism. Evidence from systematic review suggests continuity of care is associated with lower mortality (14). AWS makes continuity operational rather than aspirational: a Named Guardian is recorded, and services can report simple continuity measures (e.g., proportion with a named lead; proportion of contacts within the same team) alongside harm indicators.

This equity-first framing reflects the logic of proportionate universalism: interventions should be universal but delivered with intensity proportionate to need, recognizing steep social gradients in health risk and outcomes. Work on health equity in England underlines the importance of this approach for narrowing avoidable gradients (15). AWS offers a practical structure through which proportionate universalism can be applied to elective waiting, rather than waiting functioning as a passive amplifier of inequality.

## Discussion

The case for an Active Waiting Standard is a patient-safety and quality argument. A large, predictable interval exists between referral and first specialist contact. The system currently treats it as administratively active but clinically under-designed. AWS proposes that the interval should be explicitly recognized as a managed phase of care, with transparent ownership, inclusive access, minimal monitoring, and defined escalation.

A predictable objection is workload. Yet much of what AWS formalizes already occurs informally: repeated patient contacts seeking reassurance, unstructured medication escalation, reactive safety-netting, and ad hoc attempts to obtain advice-and-guidance. Standardizing “holding care” may reduce failure demand by detecting deterioration earlier and preventing crises that generate urgent work. It may also improve the efficiency of first specialist appointments by ensuring baseline status, trajectory, risks, and patient priorities are visible rather than reconstructed under time pressure.

Governance is crucial. AWS clarifies responsibility without implying that primary care should substitute for specialist assessment. In practical terms, this means that the referring GP will usually be the default Named Guardian, with delegation to another suitably skilled clinician used only where team-based pathways already provide clear access routes, escalation logic, and documented responsibility. Ownership is explicitly time-limited and persists until clinical takeover is confirmed, consistent with professional expectations regarding accepted responsibility during transitions (5). Crucially, AWS does not redefine clinical thresholds for urgent assessment and does not ask primary care to deliver specialist diagnostics; it standardizes monitoring, safety-netting, and escalation during the waiting phase. At system level, AWS can be implemented through simple enablers: an electronic field for Named Guardian, a visible contact route with response standards, baseline prompts, risk-banded check-ins, and agreed escalation routes supported by advice-and-guidance (6, 9).

Implementation is most feasible when it starts small. Piloting AWS in one or two high-volume streams allows rapid iteration, staff training, and pragmatic evaluation. Early specialty engagement is important so escalation routes remain credible and coordinated. Equity-first adoption should be embedded from the start, using stratified wait data to target mitigations to those most exposed to harm while waiting (4, 12, 13).

Success should be judged by outcomes during the wait: reduced deterioration, fewer emergency presentations and urgent appointments, fewer harmful medication escalations, improved patient understanding and confidence, and better productivity at first specialist contact. Equity should be evidenced as reduced harm gradients across deprivation and ethnicity, not simply as small changes in median waits.

Finally, AWS should be a platform for constructive evaluation rather than a rigid doctrine. Which elements deliver the greatest harm reduction per unit effort? What risk-banding is most efficient and equitable? How should advice-and-guidance capacity be aligned to support dynamic holding care? These questions are testable once the interval is recognized as a phase of care rather than a blank space between organizations.

In summary, elective waiting is currently treated as a queue. It should be treated as a managed phase of care. A minimum, auditable Active Waiting Standard—aligned with system priorities and elective reform, and designed to reduce inequity—offers a pragmatic route to making waiting safer and more clinically meaningful for patients and clinicians alike.
